# Radiolabeled cCPE Peptides for SPECT Imaging of Claudin-4 Overexpression in Pancreatic Cancer

**DOI:** 10.2967/jnumed.120.243113

**Published:** 2020-12

**Authors:** Julia Baguña Torres, Michael Mosley, Sofia Koustoulidou, Samantha Hopkins, Stefan Knapp, Apirat Chaikuad, Masuo Kondoh, Keisuke Tachibana, Veerle Kersemans, Bart Cornelissen

**Affiliations:** 1 Cancer Research United Kingdom and Medical Research Council Oxford Institute for Radiation Oncology, University of Oxford, Oxford, United Kingdom; 2 Institute of Pharmaceutical Chemistry and Structure Genomics Consortium, Goethe-University Frankfurt, Frankfurt am Main, Germany; 3 German Cancer Network, Mainz-Frankfurt, Germany; and; 4 Graduate School of Pharmaceutical Sciences, Osaka University, Yamadaoka, Suita, Osaka, Japan

**Keywords:** claudin-4, pancreatic ductal adenocarcinoma, early diagnosis, SPECT imaging

## Abstract

Overexpression of tight-junction protein claudin-4 has been detected in primary and metastatic pancreatic cancer tissue and is associated with better prognosis in patients. Noninvasive measurement of claudin-4 expression by imaging methods could provide a means for accelerating detection and stratifying patients into risk groups. *Clostridium perfringens* enterotoxin (CPE) is a natural ligand for claudin-4 and holds potential as a targeting vector for molecular imaging of claudin-4 overexpression. A glutathione S-transferases (GST)–tagged version of the C terminus of CPE (cCPE) was previously used to delineate claudin-4 overexpression by SPECT but showed modest binding affinity and slow blood clearance in vivo. **Methods:** On the basis of the crystal structure of cCPE, a series of smaller cCPE_194–319_ mutants with putatively improved binding affinity for claudin-4 was generated by site-directed mutagenesis. All peptides were conjugated site-specifically on a C-terminal cysteine using maleimide-diethylenetriamine pentaacetate to enable radiolabeling with ^111^In. The binding affinity of all radioconjugates was evaluated in claudin-4–expressing PSN-1 cells and HT1080-negative controls. The specificity of all cCPE mutants to claudin-4 was assessed in HT1080 cells stably transfected with claudin-4. SPECT/CT imaging of BALB/c nude mice bearing PSN-1 or HT1080 tumor xenografts was performed to determine the claudin-4–targeting ability of these peptides in vivo. **Results:** Uptake of all cCPE-based radioconjugates was significantly higher in PSN-1 cells than in HT1080-negative controls. All peptides showed a marked improvement in affinity for claudin-4 in vitro when compared with previously reported values (dissociation constant: 2.2 ± 0.8, 3 ± 0.1, 4.2 ± 0.5, 10 ± 0.9, and 9.7 ± 0.7 nM). Blood clearance of [^111^In]In-cCPE_194–319_, as measured by SPECT, was considerably faster than that of [^111^In]In-cCPE.GST (half-life, <1 min). All radiopeptides showed significantly higher accumulation in PSN-1 xenografts than in HT1080 tumors at 90 min after injection of the tracer ([^111^In]In-cCPE_194–319_, 2.7 ± 0.8 vs. 0.4 ± 0.1 percentage injected dose per gram [%ID/g], *P* < 0.001; [^111^In]In-S313A, 2.3 ± 0.9 vs. 0.5 ± 0.1 %ID/g, *P* < 0.01; [^111^In]In-S307A + N309A + S313A, 2 ± 0.4 vs. 0.3 ± 0.1 %ID/g, *P* < 0.01; [^111^In]In-D284A, 2 ± 0.2 vs. 0.7 ± 0.1 %ID/g, *P* < 0.05; [^111^In]In-L254F + K257D, 6.3 ± 0.9 vs. 0.7 ± 0.2 %ID/g, *P* < 0.001). **Conclusion:** These optimized cCPE-based SPECT imaging agents show great promise as claudin-4–targeting vectors for in vivo imaging of claudin-4 overexpression in pancreatic cancer.

## Visual Abstract

**Figure:**
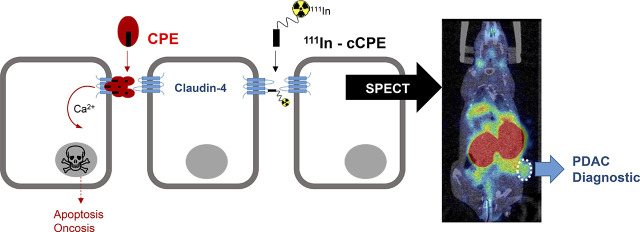

Pancreatic ductal adenocarcinoma (PDAC) is predicted to become the second-leading cause of cancer-related death within the next decade, with current 5-y survival rates reaching less than 5%. This poor prognosis is partly due to late diagnosis of this mostly nonsymptomatic disease at the metastatic stage, and mostly in the emergency medicine setting (1,2). Currently, the best probability for long-term survival is surgical resection. However, this is possible only when the disease is still in its early stages and confined to the pancreas.

Radionuclide-based imaging agents for SPECT and PET are exquisitely sensitive tools, capable of detecting molecular markers associated with malignant tissue in vivo. The use of these targeted molecular probes has the potential to greatly improve the diagnosis of pancreatic cancer by enabling the detection of neoplastic transformation and providing functional and prognostic information about the disease. Radiotracers such as the fluorinated glucose analog ^18^F-FDG have proved able to detect PDAC, distinguish benign mass-forming lesions, and exclude patients with multiple and distant occult metastatic disease from nonbeneficial surgery (3). However, the use of ^18^F-FDG PET as an early diagnostic test is hampered by an inability to distinguish PDAC from chronic or autoimmune pancreatitis (4,5) or for evaluation in PDAC during chemo- or radiochemotherapy (6,7). Use of other PET imaging agents in pancreatic cancer patients, such as ^18^F-fluoromisonidazole and ^18^F-fluoro-3′-deoxy-3′-l-fluorothymidine, have produced mixed results, highlighting the need for alternative tools to assess prognosis and treatment response (8,9). Targeting more disease-specific molecular markers could therefore considerably aid in the early detection and characterization of pancreatic cancer lesions.

Claudin transmembrane proteins are considered the structural and functional backbone of tight-junction complexes in epithelial and endothelial cells (10–12). As tumorigenesis is often accompanied by tight-junction disruption and subsequent loss of cell adhesion and polarity, it is not surprising that expression of several claudins is dysregulated in many cancers of epithelial origin. In particular, claudin-4 has been shown to be overexpressed in primary and metastatic pancreatic cancer tissue, including pancreatic intraepithelial neoplasia (PanIN), the most common precursor lesion to PDAC (13). Unlike previous biomarkers, increased expression of claudin-4 has not been reported in chronic pancreatitis, which makes this protein an attractive target for early detection of PDAC (14). Furthermore, claudin-4 overexpression has been identified as a potent inhibitor of the invasive and metastatic phenotypes of pancreatic cancer cells and correlates with better outcome in PDAC patients (15,16). Knockdown studies in ovarian cancer cells suggest that this positive effect of claudin-4 overexpression may be due to its ability to sustain the expression of E-cadherin and limit β-catenin signaling (17,18). Hence, claudin-4 can be regarded not only as a promising biomarker for early detection of PDAC but also as a cancer type–specific prognostic indicator.

*Clostridium perfringens* enterotoxin (CPE, 35 kDa), a natural ligand for claudin-4, has been previously evaluated as a claudin-4–targeting vector for diagnostic and therapeutic purposes. CPE causes the symptoms of type A food poisoning and some non–food-borne gastrointestinal diseases by triggering the lysis of intestinal epithelial cells through its interaction with claudin-4 receptors. Notably, CPE cytotoxicity is mediated by its N terminus (NH_2_-CPE, amino acids 1–183), whereas the C-terminal portion (cCPE, amino acids 184–319, 17 kDa) is responsible for interaction with claudin-4 (19,20). Taking advantage of the claudin-binding activity of full-length cCPE, Neesse et al. fused this fragment to glutathione S-transferase (cCPE.GST, 41 kDa) and fluorophore Cy5.5 to visualize claudin-4 overexpression in tumor xenograft and genetically engineered mouse models of pancreatic cancer by near-infrared imaging (14). Similarly, Cocco et al. used a fluorescein isothiocyanate–conjugated cCPE peptide to successfully measure claudin-4 levels in mouse models of ovarian cancer (21). To overcome the limitations associated with optical imaging, as well as antibody-mediated imaging probes (22), our group labeled a cCPE.GST conjugate with the γ-emitting radioisotope ^111^In (half-life, 2.8 d) to enable noninvasive whole-body claudin-4 detection in breast cancer mouse models by SPECT imaging. This study confirmed the ability of [^111^In]In-cCPE.GST to delineate claudin-4 expression both in overt mammary tumors and in aplastic lesions in genetically engineered BALB/neuT mice (23).

Despite its promise as an early detection tool for claudin-4–overexpressing malignancies, [^111^In]In-cCPE.GST showed major drawbacks that limit its general utility, such as a moderate claudin-4 binding affinity (in the micromolar range), long circulation half-life, and undetermined specificity. In previous studies, a shift in claudin-4 binding properties of the cCPE vector was achieved through structure-based modifications of the C-terminal 223–319 amino acid region, through a series of point mutations (24,25). Here, we generated a series of smaller mutant cCPE-based radiotracers with the aim of improving the pharmacokinetics and claudin-4–targeting ability of wild-type cCPE and evaluated these using in vitro and in vivo mouse models of PDAC.

## MATERIALS AND METHODS

### Reagents and Cell Lines

Rabbit polyclonal (PA5-28830) and mouse monoclonal (329400) anticlaudin-4 and rabbit polyclonal anticlaudin-3 (341700) antibodies were purchased from ThermoFisher Scientific. Goat antimouse horseradish-peroxidase–conjugated (5178-2504) and goat antirabbit IgG Alexa Fluor 488 (A11034) antibodies were obtained from Bio-Rad and Invitrogen, respectively. The chelating agent maleimide-diethylenetriamine pentaacetate (DTPA) was purchased from CheMatec. Water was deionized using a Barnstead NANOpure purification system (Thermo Scientific) and had a resistance of more than 18.2 MΩ/cm at 25°C. Protein concentration was measured on a ND-1000 spectrophotometer (NanoDrop Technologies, Inc.). Instant thin-layer chromatography was performed on glass microfiber chromatography paper (Agilent Technologies), and strips were analyzed with either a Bioscan AR-2000 radio-thin-layer chromatography scanner (Eckert and Ziegler) or a Cyclone Plus Phosphor Imager (PerkinElmer). Radioactivity measurements were determined using a CRC-25R dose calibrator (Capintec, Inc.).

PSN-1 (human pancreatic adenocarcinoma) and HT1080 (human connective tissue epithelial fibrosarcoma) cell lines were obtained from the American Type Culture Collection. Each of these cell lines was maintained in Dulbecco modified Eagle medium, supplemented with 10% fetal bovine serum, 2 mM l-glutamine, 100 units/mL penicillin, and 0.1 mg/mL streptomycin. HT1080 cells that were stably transfected with human claudin-4 (HT1080/hCLDN-4) were generated as previously described (26) and cultured in Dulbecco modified Eagle medium (10% fetal bovine serum, 2 mM l-glutamine, 100 units/mL penicillin, and 0.1 mg/mL streptomycin) supplemented with 5 μg/mL puromycin and 500 μg/mL zeocin. All cells were cultured in a 37°C environment containing 5% CO_2_. Cells were harvested and passaged as required using trypsin-ethylenediaminetetraacetic acid solution. Cells were tested and authenticated by the providers. The cumulative length of culture was less than 6 mo after retrieval from liquid nitrogen storage. Cell were tested for the absence of *Mycoplasma* at regular intervals.

### Western Blotting

Total cell lysis was performed at 4°C on approximately 1 × 10^7^ cells using radioimmunoprecipitation assay lysis buffer (50 mM Tris, pH 8, 1% NP40, 0.5% sodium deoxycholate, 0.1% sodium dodecyl sulphate, 150 mM sodium chloride, cOmplete protease inhibitor cocktail [Sigma-Aldrich]). Cell lysates were isolated by centrifugation after lysis through a 21-gauge hypodermic syringe and 30 s of sonication. Thirty-microgram lysate samples were run on a 4%–12% Bis-Tris MES gel (Novex), transferred to a polyvinylidene difluoride membrane, and exposed to a 1:700 dilution of anticlaudin-4 antibody (329400; ThermoFisher Scientific), followed by a 1:3,000 dilution of the secondary goat antimouse–horseradish peroxidase (5178-2504; Bio-Rad). The blots were developed using Pierce ECL Western Blotting Substrate (32106; ThermoFisher Scientific) and exposed to a Li-Cor 3600 Blot scanner.

### Immunofluorescence Staining

PSN-1, HT1080, or HT1080/hCLDN4 cells (70,000 cells per well) were seeded onto 8-chamber glass slides (Falcon) and allowed to adhere overnight. Cells or frozen tumor xenograft sections (10 μm) were washed in phosphate-buffered saline and fixed using 4% paraformaldehyde solution (10 min). After washing, the sections were permeabilized using 0.5% digitonin for 15 min at room temperature, washed again, and blocked in 2% bovine serum albumen for 1 h at room temperature. The cells or sections were then exposed to anticlaudin-4 (PA5–28830; 1:100) or anticlaudin-3 (341700; 1:100) antibody at 4°C overnight, washed in phosphate-buffered saline (3 × 5 min), and incubated with goat antirabbit IgG Alexa Fluor 488 (1:500) for 1 h at room temperature. After further washing (3 × 5 min), the slides were mounted using Vectashield with 4′,6-diamidino-2-phenylindole (Vector H-1200). Fluorescence micrographs were acquired using a TCS SP8 confocal microscope (Leica Microsystems).

### Peptide Expression, Modification, and Radiolabeling

A C-terminal fragment of CPE (amino acids 194–319) linked to an H10 fusion protein (H_10_-cCPE_194–319_) has previously been produced and supplied by Dr. Yasuhiko Horiguchi (27). H_10_-cCPE_194–319_ was used as a template for standard site-directed mutagenesis methodologies (the supplemental information provides additional detail) to make specific amino acid substitutions, which were verified by sequencing of the resulting plasmid (Source BioScience). The modified peptides were expressed in *E. coli* by isopropyl β-d-1-thiogalactopyranoside (MP Biomedicals LLC) addition and purified by affinity chromatography using HisLink protein purification resin (Promega). A detailed procedure is laid out in the supplemental data. Purified proteins were dialyzed against phosphate-buffered saline/2 mM ethylenediaminetetraacetic acid at 4°C overnight. Purity of the peptides was confirmed by mass spectroscopy and sodium dodecyl sulfate and polyacrylamide gel electrophoresis gel followed by Coomassie blue staining.

Purified cCPE peptides were concentrated to 2 mg/mL using an ultrafiltration filter device (Amicon, 3-kDa molecular-weight size cutoff; Merck Millipore Ltd.) and incubated with tris(2-carboxyethyl)phosphine hydrochloride (4 mg/mL in water, 3 equivalents; Cayman Chemical Co.) at 25°C, agitating at 300 rpm for 90 min. The peptides were then reacted with maleimide-DTPA (10 mg/mL in dimethyl sulfoxide, 5 equivalents; CheMatech) at 25°C and 300 rpm for 90 min and purified using a Sephadex G-25 resin (Sigma-Aldrich) in sodium citrate buffer (0.1 M, pH 5.5). DTPA-conjugated peptides were concentrated by centrifugation using 3-kDa centrifugal filter units (Millipore).

^111^In in 0.02 M hydrochloric acid (Curium Pharma U.K. Ltd.) was added to a 2 mg/mL solution of the DTPA-conjugated cCPE peptides to achieve a 0.1–1 MBq/μg molar activity (5.9–59 × 10^−9^ GBq/μmol). The reaction mixture was incubated at 37°C for 90 min, and the radiolabeling efficiency was determined by instant thin-layer chromatography using an eluent of 50 mM ethylenediaminetetraacetic acid (pH 5.5). When purification was required, the crude reaction mixture was purified by Sephadex-G25 size-exclusion chromatography in phosphate-buffered saline (pH 7.4).

### Cell-Binding Assays

To investigate binding of [^111^In]In-cCPE peptides to claudin-4 receptors, aliquots of 1.5 × 10^5^ PSN-1, expressing claudin-4; HT1080, not expressing claudin-3 or -4; and HT1080/hCLDN4, expressing only claudin-4, were seeded onto a 24-well plate in 500 μL of growth medium and allowed to adhere overnight. Cells were then incubated with increasing amounts (0.5–160 nM) of [^111^In]In-DTPA-cCPE (1 MBq/μg) for 2 h at 4°C. After incubation, supernatant was removed and cells were washed and lysed using 0.1 M sodium hydroxide. ^111^In radioactivity in the cell-associated fractions was measured using an automated γ-counter (Wizard^2^ 2480; Perkin Elmer) and normalized by the amount of protein present in the seeding controls determined by bicinchoninic acid assay. Binding affinity (dissociation constant [K_D_]) was estimated by nonlinear regression analysis with a 1-site total binding model using the software package Prism (version 7; GraphPad Software Inc).

### In Vivo Studies

All animal procedures were performed in accordance with the U.K. Animals (Scientific Procedures) Act of 1986 and with local ethical committee approval.

In vivo imaging experiments were performed using a VECTor^4^CT SPECT/CT system (MILabs). For dynamic SPECT imaging, male BALB/c mice (*n* = 3) were anesthetized by 4% isoflurane gas (0.5 L/min O_2_) and placed prone on a custom‐built imaging cradle. Animals were intravenously injected with [^111^In]In-cCPE_194–319_ (5 μg, 5 MBq, < 200 μL) through a tail vein catheter, and dynamic imaging was performed over 90 min after tracer administration. SPECT data were acquired (150 frames, 30 s per frame, 2.1 s/bed position using list-mode acquisition) using an ultra‐high-resolution rat-and-mouse 1.8-mm collimator, followed by a cone‐beam CT scan (55 kV, 0.19 mA, 20 ms) for anatomic reference and attenuation correction. Anesthesia was maintained at 2.5% isoflurane, and body temperature was kept at 37°C throughout the scan. SPECT images were reconstructed using U‐SPECT‐Rec3.22 software (MILabs), applying a pixel‐based algorithm with 8 subsets, 6 iterations, and a 0.8 mm^3^ voxel size for ^111^In (energy window, 159–278 keV; background weight, 2.5).

To allow quantification of SPECT data, calibration factors derived from ^111^In phantoms were used. SPECT images were registered to their corresponding CT images and attenuation-corrected. SPECT images were quantified using volume-of-interest analyses with the PMOD software package (version 3.807; PMOD Technologies) to calculate the percentage injected dose per milliliter of volume of interest (%ID/mL) per time bin.

After imaging, the mice were euthanized by cervical dislocation, and organs of interest were excised and weighed. The amount of radioactivity in each organ was measured using a WIZARD^2^ 2470 γ-counter (PerkinElmer). Counts per minute were converted to megabecquerels using a calibration curve generated from known standards. All values were decay-corrected to the time of injection, and the percentage injected dose per gram (%ID/g) of each sample was calculated.

Tumors were generated in BALB/c *nu/nu* mice by subcutaneous injection of 10^6^ cells in Dulbecco modified Eagle medium. Tumors were allowed to grow for 3–4 wk. Animals were used for subsequent studies when the tumors reached 200 mm^3^. Static SPECT images were acquired after intravenous administration of ^111^In-labeled cCPE peptides (5 μg, 5 MBq) in sterile 0.9% saline (<200 μL). The peptides were injected intravenously via the lateral tail vein (*n* = 3/peptide/cell line). SPECT/CT images were acquired at 90 min after injection of the tracer over 7 min using a 1.8-mm pinhole rat collimator (3 frames, 2.3 min/frame, 11.5 s/bed position).

### Ex Vivo

After imaging, tumor xenografts were flash-frozen in isopentane (Sigma-Aldrich), chilled with liquid nitrogen, and stored at −80°C overnight. Frozen tissue was sectioned (10 μm) using an OTF5000 cryotome (Bright Instruments Ltd.). Tissue sections were thaw-mounted onto SuperFrost Plus glass microscope slides (Menzel-Glaser, Thermo Scientific) and allowed to dry at room temperature. The slides were then exposed to a storage phosphor screen (Super Resolution, 12.5 × 25.2 cm; PerkinElmer) for 15 h. The phosphor screen was then imaged using a Cyclone Plus Storage Phosphor System.

### Statistical Analyses

All statistical analyses and nonlinear regression were performed using Prism. One-way ANOVA was used for multiple comparisons, with Tukey posttests to calculate the significance of differences between groups. All data were obtained as triplicate independent replicates. Results are reported and graphed as average ± SD, unless stated otherwise.

## RESULTS

### Claudin-4 Expression in Human Pancreatic Cancer Cell Lines

**FIGURE 1.**
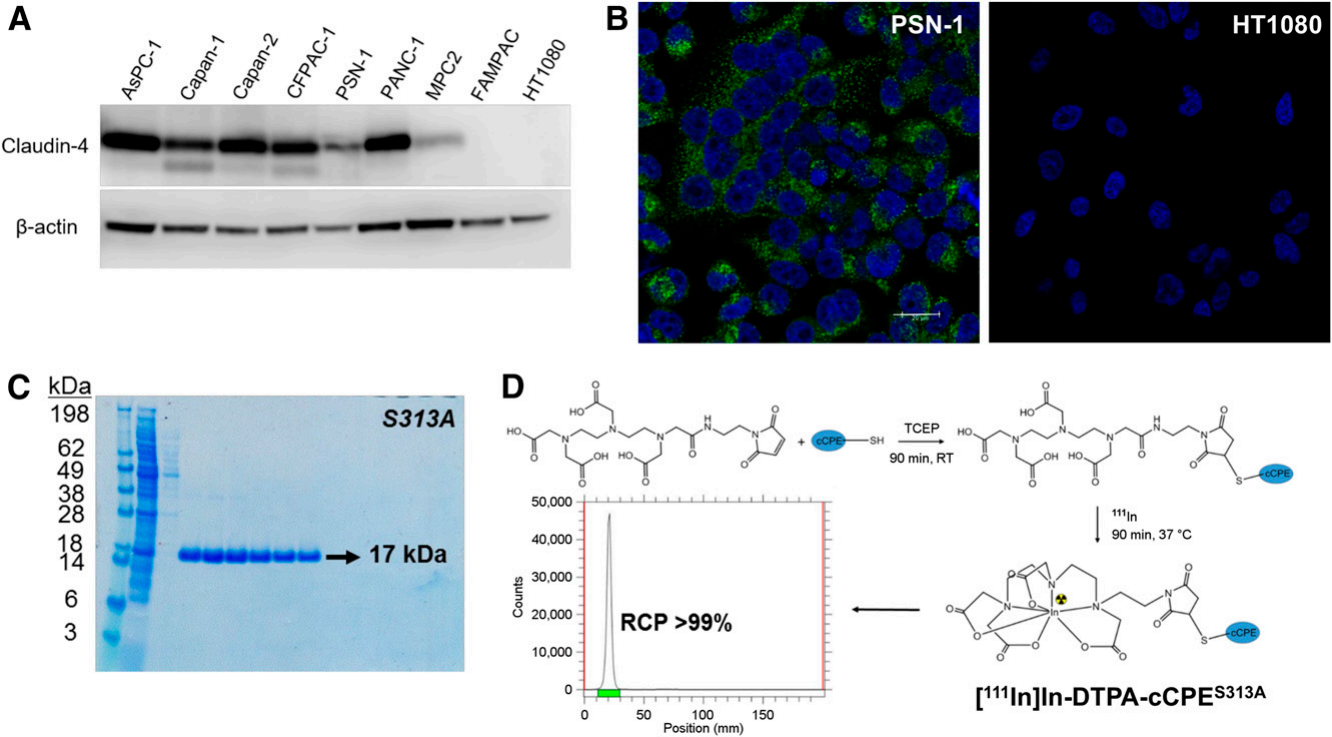
(A) Claudin-4 expression in panel of human pancreatic cancer cell lines detected by Western blot. (B) Immunofluorescence staining of claudin-4 expression in human PDAC PSN-1 cell line and claudin-4–nonexpressing HT1080 fibrosarcoma cells (green: claudin-4; blue: 4′,6-diamidino-2-phenylindole, representing cell nuclei; scale bar is 20 μm). (C) Coomassie blue–stained sodium dodecyl sulfate and polyacrylamide gel electrophoresis gel of purified S313A variant. All peptides were obtained with >95% purity. (D) Schematic representation of bioconjugation and ^111^In radiolabeling strategies for cCPE mutants. cCPE peptides were initially reduced with tris(2-carboxyethyl)phosphine hydrochloride and site-specifically conjugated to maleimide-DTPA. Resulting bioconjugates were radiolabeled with ^111^In, with excellent radiochemical purity (>99%) and yield (>95%). RCP = radiochemical purity; RT = room temperature; TCEP = tris(2-carboxyethyl)phosphine hydrochloride.

Eight pancreatic cell lines of human origin were analyzed by Western blot for claudin-4 expression (Fig. 1A). Claudin-4 was found to be expressed in 7 of 8 cell lines, with the highest levels in the human pancreatic adenocarcinoma Capan-2 cells and the lowest in the human pancreatic epithelial carcinoma Mia-PaCa-2 cells. No claudin-4 expression was detectable by Western blot in the human pancreatic carcinoma FAMPAC-1 cells or the human fibrosarcoma HT1080 cell line. The latter served as a negative control in this study. Because of their fast growth rate and ability to establish robust and reproducible subcutaneous tumor xenografts in mice, PSN-1 cells were selected among all claudin-4–expressing PDAC cell lines for further experiments (28).

The presence of claudin-4 in PSN-1 cells was also confirmed by fluorescence immunocytochemistry (Fig. 1B). Claudin-4 protein was found to be localized at the cell–cell contacts and associated with intracellular vesicles in confluent PSN-1 cells but not in HT1080 cells. In PDAC sections harvested from patients, claudin-4 is expressed strongly at the cell membrane (29).

### Design and Synthesis of cCPE-Based Radiotracers

To improve the pharmacokinetics and claudin-4–binding properties of [^111^In]In-cCPE.GST, 5 smaller variants were generated, using structural proteomic and recombinant protein expression methodologies (Supplemental Fig. 1) (*18*). On the basis of the crystal structure of cCPE, and the knowledge that residues 290–319 are necessary for claudin binding, specific amino acid substitutions were introduced to the wild-type sequence (amino acids 194–319) by site-directed mutagenesis in order to enhance the affinity (S313A and S307A + N309A + S313A) or specificity (D284A and L254F + K257D) of cCPE for claudin-4 (20,30). Although some mutations had previously been reported in the literature, the L254F + K257D modification was predicted through molecular docking modeling of the interaction between the cCPE binding site, claudin-4, and several closely related claudins (claudin-3 and claudin-19) (Supplemental Fig. 1) (24,25). Additionally, a single cysteine residue was added to the sequence to allow site-specific conjugation of the peptide to a maleimide-DTPA chelator. Furthermore, 10 amino acids of wild-type cCPE (amino acids 184–193) were removed from the vector as they were found not to be crucial for claudin-4 targeting (20). Together, these changes led to a reduction in size from 41 to 17 kDa, with the aim to produce more rapid pharmacokinetics and allow faster imaging. A plasmid vector expressing the cCPE_194–319_ fragment linked to an H10 fusion protein for purification, instead of the far larger GST fusion protein, was used as a template for modifications induced by site-directed mutagenesis—modifications that were verified by sequencing (Supplemental Fig. 1).

All plasmids, encoding for wild-type or mutant cCPE.histidine (his)10 sequences, were introduced into competent *E. coli* BL21(DE3) cells. Protein expression was induced via isopropyl β-d-1-thiogalactopyranoside addition, and the resulting peptides were purified by nickel affinity chromatography and dialysis (Fig. 1C) and characterized by mass spectroscopy (Supplemental Fig. 2). After modification with maleimide-DTPA, all cCPE peptides were radiolabeled with ^111^In with excellent radiochemical yield (>99%) and purity (>99%) and a typical molar activity of 1 MBq/μg (Fig. 1D).

### In Vitro Characterization

**FIGURE 2.**
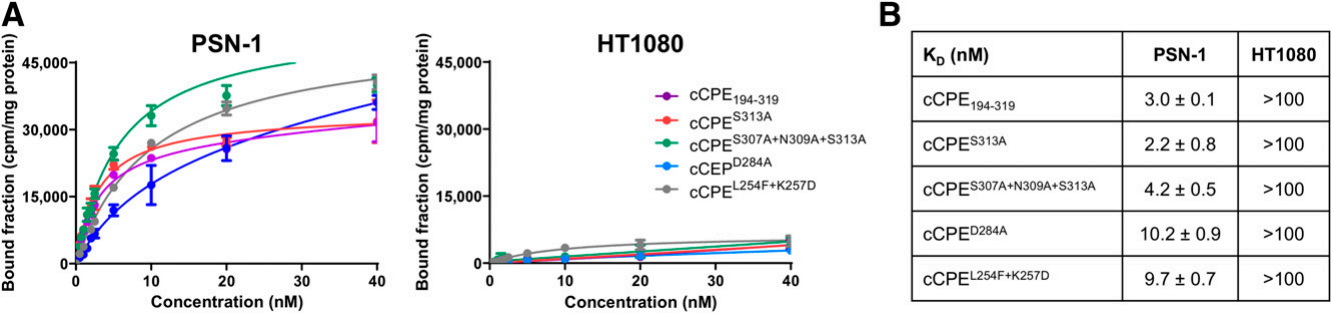
(A) PSN-1 and HT1080 cells were exposed for 2 h at 4°C to increasing concentrations of ^111^In-labeled cCPE peptides, and extent of cell binding was determined. (B) Experimental results for all tested cCPE radiopeptides in PSN-1 and HT1080 cells.

All radiolabeled peptides bound claudin-4–expressing PSN-1 cells in a saturable manner, but not claudin-4–negative HT1080 control cells (Fig. 2A). After exposure of cells to radiolabeled cCPE peptides at a saturation concentration (40 nM) for 2 h, radioactivity associated with PSN-1 cells was approximately 9 times higher than that associated with HT1080 cells (*P* < 0.0001). Saturation binding assays yielded affinities (K_D_) in the nanomolar range (2.2–10.2 nM) for all labeled cCPE-based peptides, a marked improvement over earlier reported values in the micromolar range for unmodified CPE binding to purified His_10_-claudin-4 or [^111^In]In-cCPE.GST binding to claudin-4–expressing human mammary gland/breast epithelial MDA-MB-468 cells (0.65 and 1.93 μM, respectively (14,23)). The wild-type cCPE_194–319_, S313A, and S307A + N309A + S313A variants showed the better binding affinity in PSN-1 cells (K_D_: 2.2 ± 0.8, 3 ± 0.1, and 4.2 ± 0.5 nM, respectively), whereas the D284A and L254F + K257D mutants showed a higher K_D_ in PSN-1 cells (10 ± 0.9 and 9.7 ± 0.7 nM, respectively) (Fig. 2B).

**FIGURE 3.**
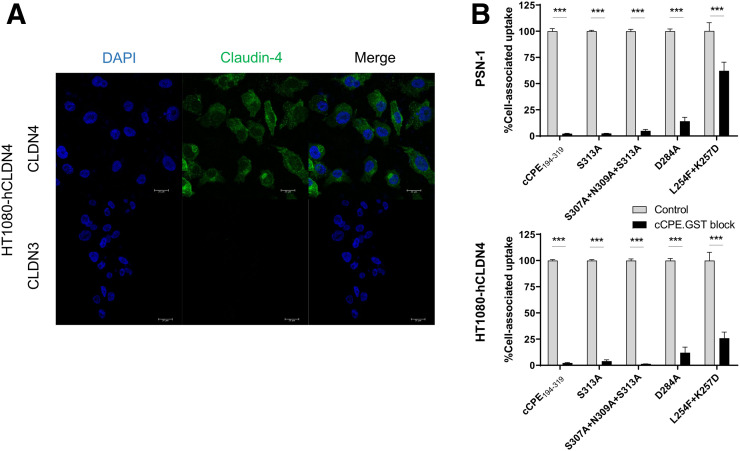
(A) Immunocytochemistry of claudin-4 (top) and claudin-3 (bottom) in HT1080 cells stably transfected with human claudin-4 (HT1080-hCLDN4). Green fluorescence indicates claudin-4–positive staining, and blue (4′,6-diamidino-2-phenylindole [DAPI]) fluorescence indicates cell nuclei. Scale bar is 20 μm. (B) An excess of cold, unlabeled cCPE.GST (100-fold) was used to block binding of cCPE radiopeptides to PSN-1 and HT1080-hCLDN4 cells. ***P < 0.001.

To evaluate the specificity of these radiolabeled cCPE peptides for claudin-4 receptors, we performed in vitro blocking studies by adding increasing amounts of cold, unlabeled cCPE.GST (100-fold) (Fig. 3). In addition to PSN-1 cells, we used the HT1080-hCLDN4 cell line, stably transfected with human claudin-4 and lacking expression of claudin-3, a closely related claudin protein. We confirmed strong vesicular and membrane claudin-4 immunostaining but absence of claudin-3 signal in HT1080-hCLDN4 cells (Fig. 3). Efficient blocking (>95%, all *P* < 0.001) of ^111^In-labeled cCPE_194–319_, S313A, and S307A + N309A + S313A was achieved by addition of a 100-fold excess of cCPE.GST in both PSN-1 and HT1080-hCLDN4, suggesting a highly specific interaction of these cCPE variants and claudin-4, and other potential binding epitopes of cCPE, with very little nonspecific uptake. Cell-associated uptake of [^111^In]In-cCPE^D284A^ could be blocked only by 85% in both cell lines (*P* < 0.001), whereas the ^111^In-cCPE^L254F+K257D^ mutant showed the lowest specificity of all peptides, with only 38% blocking achieved in PSN-1 cells and 74% in HT1080-hCLDN4 cells (*P* < 0.001).

For additional cross-validation of the radiolabeled cCPE^S313A^, this variant was also fluorescently labeled using a Cy5 maleimide dye. PSN-1 cells were exposed to Cy5-labeled cCPE^S313A^ and subsequently imaged by confocal microscopy (Supplemental Fig. 3). The fluorescence associated with Cy5-cCPE^S313A^ was localized mainly in the cytoplasm and plasma membrane of PSN-1 cells, similar to claudin-4 immunostaining.

### In Vivo Imaging

Initial in vivo evaluation of our tracer design was performed in wild-type mice using the ^111^In-radiolabeled wild-type cCPE_194–319_ variant to determine pharmacokinetics. Otherwise naïve BALB/c mice were injected intravenously with the tracer via a tail vein catheter and imaged dynamically by SPECT over 75 min. The radiopeptide cleared rapidly from blood, after a monoexponential decay pattern with a half-life of 0.5 ± 0.2 min, as measured by image volume-of-interest analysis on kinetic SPECT images (Supplemental Fig. 4). Both SPECT imaging and ex vivo γ-counting revealed a high accumulation of the radiopeptide in the kidneys, liver, and salivary glands (32.9 ± 6.7, 25.3 ± 5.8, and 6.3 ± 2.1 %ID/mL, respectively, at 75 min after injection). Elimination of [^111^In]In-cCPE_194–319_ occurred through renal and hepatic routes.

**FIGURE 4.**
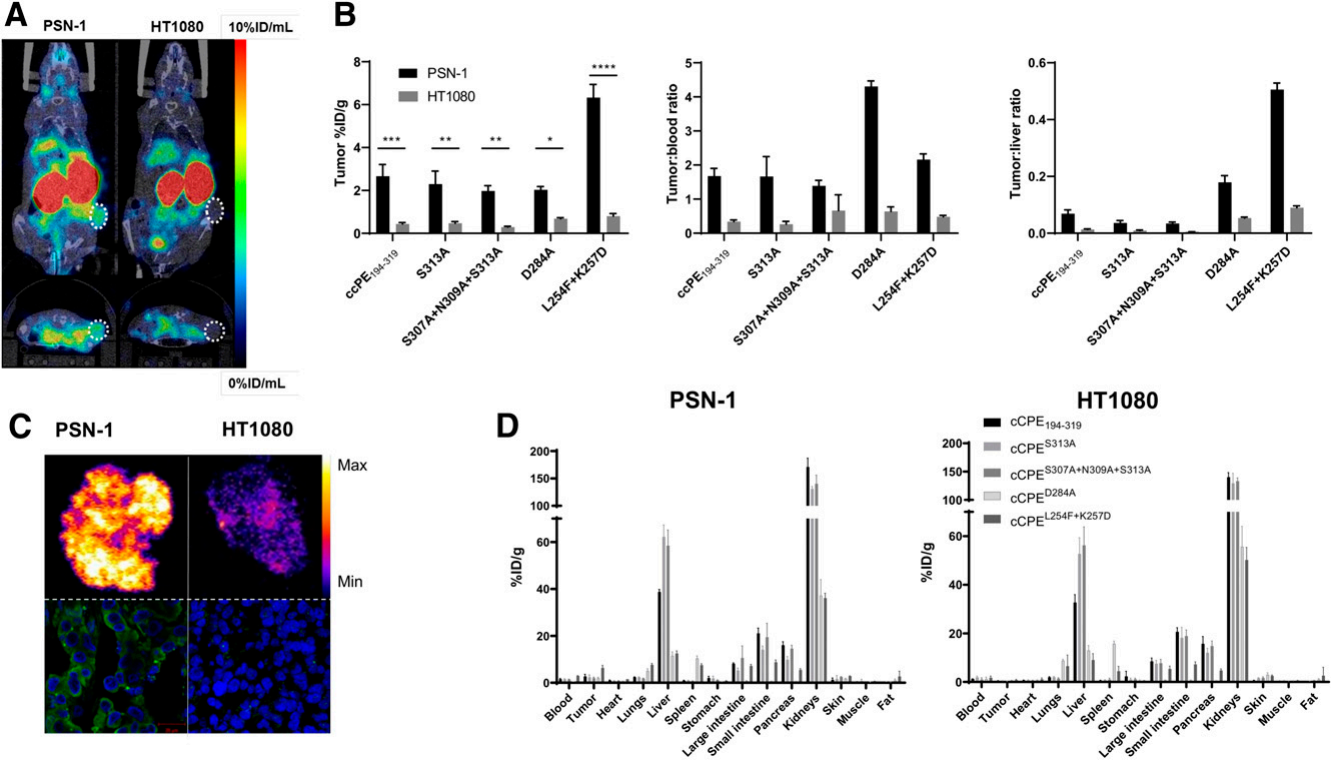
(A) Representative SPECT/CT images of mice carrying tumor xenografts (white circles) of PSN-1 (claudin-4–positive) or HT1080 (claudin-4–negative) cells 90 min after intravenous injection of [^111^In]In-cCPE^L254F+K257D^. Coronal and axial sections through tumor are shown. (B) Ex vivo tumor uptake (%ID/g), tumor-to-blood ratios, and tumor-to-liver ratios of ^111^In-radiolabeled cCPE peptides 2 h after intravenous administration of tracer. *P < 0.05. **P < 0.01. ***P < 0.001. (C) Autoradiography images of PSN-1 and HT1080 tumor xenograft sections of mice injected with [^111^In]In-cCPE^L254F+K257D^ and corresponding confocal images of immunofluorescence staining of claudin-4 (green: claudin-4; blue: 4′,6-diamidino-2-phenylindole; scale bar is 20 μm). PSN-1 tumor sections showed significantly higher tracer uptake than negative control. (D) Biodistribution of ^111^In-radiolabeled cCPE peptides 2 h after intravenous administration of tracer.

The feasibility of claudin-4–targeted tumor detection in vivo with wild-type versus mutant cCPE-based radiotracers was then assessed in immunocompromised mice bearing subcutaneous PSN-1 or HT1080 tumor xenografts (Fig. 4). All mice were injected intravenously with cCPE radiotracer (*n* = 3/peptide per cell line) and imaged by SPECT/CT at 90 min after administration, to ensure that most radioactivity had cleared from blood. Ex vivo counting of organs and tissues at 90 min after injection showed a significantly higher accumulation of all cCPE-based radiotracers in claudin-4–expressing PSN-1 tumor xenografts than in HT1080 controls ([^111^In]In-cCPE_194–319_: 2.7 ± 0.8 vs. 0.4 ± 0.1 %ID/g, *P* < 0.001; [^111^In]In-cCPE^S313A^: 2.3 ± 0.9 vs. 0.5 ± 0.1 %ID/g, *P* < 0.01; [^111^In]In-cCPE^S307A+N309A+S313A^: 2 ± 0.4 vs. 0.3 ± 0.1 %ID/g, *P* < 0.01; [^111^In]In-cCPE^D284A^: 2 ± 0.2 vs. 0.7 ± 0.1 %ID/g, *P* < 0.05; [^111^In]In-cCPE^L254F+K257D^: 6.3 ± 0.9 vs. 0.7 ± 0.2 %ID/g, *P* < 0.001) (Fig. 4B). [^111^In]In-cCPE^L254F+K257D^ exhibited the highest uptake in PSN-1 tumor tissue, nearly 3-fold greater than average tumor uptake of the other labeled compounds tested here, and similar to that previously reported for [^111^In]In-cCPE.GST in MDA-MB-468 xenografts at 24 h after administration of the tracer (6.72 ± 0.18 %ID/g) (23). Ex vivo autoradiography of xenograft sections corroborated higher tracer uptake in PSN-1 tumors than in HT1080 xenografts. Immunofluorescence imaging confirmed claudin-4 overexpression in PSN-1 but not in HT1080 tumor xenografts (Fig. 4C).

As expected, tracer clearance from blood was fast, resulting in good tumor-to-blood ratios in PSN-1 xenograft–bearing mice at 90 min after injection of the tracer, all significantly higher than for the claudin-4–negative controls except for [^111^In]In-cCPE^S307A+N309A+S313A^ ([^111^In]In-cCPE_194–319_: 1.7 ± 0.3 vs. 0.3 ± 0.1, *P* < 0.01; [^111^In]In-cCPE^S313A^: 1.7 ± 0.8 vs. 0.3 ± 0.1, *P* < 0.01; [^111^In]In-cCPE^S307A+N309A+S313A^: 1.4 ± 0.2 vs. 0.7 ± 0.7, *P* > 0.05; [^111^In]In-cCPE^D284A^: 4.3 ± 0.2 vs. 0.6 ± 0.2, *P* < 0.001; [^111^In]In-cCPE^L254F+K257D^: 2.2 ± 0.2 vs. 0.5 ± 0.1, *P* < 0.001). Tumor-to-muscle ratios were excellent for all radiopeptides, with [^111^In]In-cCPE^D284A^ and [^111^In]In-cCPE^L254F+K257D^ showing the best contrast with respect to HT1080 tumor xenografts ([^111^In]In-cCPE_194–319_: 6.2 ± 1.5 vs. 1.2 ± 0.3, *P* < 0.001; [^111^In]In-cCPE^S313A^: 3.8 ± 1.2 vs. 0.9 ± 0.2, *P* < 0.01; [^111^In]In-cCPE^S307A+N309A+S313A^: 3.4 ± 0.6 vs. 0.7 ± 0.1, *P* < 0.05; [^111^In]In-cCPE^D284A^: 7.4 ± 1 vs. 1.5 ± 0.3, *P* < 0.001; [^111^In]In-cCPE^L254F+K257D^: 10 ± 1.1 vs. 1.4 ± 0.5, *P* < 0.001). Although tracer uptake in blood and muscle was low, all radiotracers displayed prominent renal and hepatic retention in both xenograft models, which limited overall image contrast. Among all peptides, [^111^In]In-cCPE^D284A^ and [^111^In]In-cCPE^L254F+K257D^ showed the lowest accumulation in the liver (PSN-1: 11.5 ± 1.4 and 12.5 ± 1.4 %ID/g) and kidneys (PSN-1: 37.1 ± 5.5 and 36.1 ± 1.7 %ID/g) when compared with [^111^In]In-cCPE_194–319_ (liver: 38.7 ± 0.9 %ID/g; kidneys: 171.2 ± 12.9 %ID/g), [^111^In]In-cCPE^S313A^ (liver: 62.2 ± 4.2 %ID/g; kidneys: 130.9 ± 3.6 %ID/g), and [^111^In]In-cCPE^S307A+N309A+S313A^ (liver: 58.5 ± 5.4 %ID/g; kidneys: 140.2 ± 13 %ID/g) (Fig. 4D).

## DISCUSSION

Tight-junction dysfunction is an important hallmark of epithelial carcinomas and plays a key role in tumor growth and metastatic progression. Claudin-4 overexpression is a common signature of this process and is considered a promising biomarker for cancer detection and prognosis in a variety of cancer types. The role of claudin-4 in pathogenesis is yet to be fully elucidated; it has been targeted for molecular imaging with both anticlaudin-4 antibodies and CPE-based peptides—a means for speeding up diagnosis, stratifying patients into risk groups, and even assessing response to therapy (31,32). A claudin-4 imaging agent might also be a useful tool for monitoring the effects of claudin-4–targeted therapies, which have shown promise in suppressing tumor growth in several preclinical cancer models (31,33,34). Neesse et al. first used a fluorescently labeled cCPE.GST peptide for detection of pancreatic cancer (14), and Cocco et al. investigated fluorescently labeled small cCPE-based peptides for intraoperative imaging (21). We previously reported on the characterization of a radiolabeled anti-claudin-4 antibody (22). An in-depth validation of these claudin-targeting compounds is not always systematically performed, making comparison of characteristics more challenging. cCPE-based imaging agents offer the advantage of faster clearance rates and shorter acquisition times than are possible for anticlaudin-4 antibodies. Several studies have reported the use of this toxin fragment to measure claudin-4 levels noninvasively. We previously radiolabeled a cCPE.GST peptide with ^111^In to enable SPECT imaging of claudin-4 in mouse models of breast cancer (23). Although targeted imaging was achieved, this probe showed only modest target-binding affinity and specificity. Hence, improving the binding properties of cCPE for claudin-4 receptors is required before clinical translation.

The compounds described here, a combination of previously described empirically validated enabling mutations and de novo designed variants, show a much improved combination of pharmacokinetics and affinity over previous radiolabeled claudin-4–targeting imaging agents (22,23). The affinity of the novel radiolabeled cCPE.his_10_ proteins, measured in vitro, was consistently stronger than that of [^111^In]In-cCPE.GST in MDA-MB-468 cells, in the low nanomolar versus low micromolar range, possibly because of a different presentation of the claudin-binding domain, although there was little affinity advantage over anticlaudin-4 IgG. The lack of a bulky GST tag also resulted in far faster elimination from blood, allowing in vivo imaging after a few hours, rather than 1–3 d. Compared with an IgG, production of small cCPE proteins is far more economical. In addition, the smaller size of the his10 series may provide improved access to claudin-4, expressed in the tight spaces between cells, increasing target epitope availability. This improved access may also explain the high uptake in the intestines of the cCPE.his10 series, compared with its bulkier variants, as the cCPE.his10 series has better access to the claudin epitopes that are expressed on the luminal side of the intestinal lining.

Point mutations in the claudin epitope-binding domain significantly influenced claudin-4 affinity, although effects were minimal apart from the D284A variant. Mutational changes more profoundly influenced pharmacokinetics and selectivity. Whereas the uptake of wild-type cCPE and the S313A and triple mutants in claudin-4–expressing cell lines could be blocked with an excess of cCPE.GST, this was not true to the same extent for D284A and L254F/K257D, indicating they may have a different selectivity profile. Regarding kinetics, notably the D284A showed far faster blood clearance (with blood values at 90 min after injection up to 3-fold lower than for other compounds), resulting in consistently lower normal-tissue and intestinal uptake. Both D284A and L254F + K257D variants show lower liver accumulation. This resulted in improved tumor-to-blood and tumor-to-liver ratios. Hepatic expression of claudin-4 is low, compared with tumor, although claudin-3 expression is not insignificant (35) and may play a role in the differences we have observed. Taken together, these results highlight that small changes in the cCPE sequence can thus result in large changes in pharmacokinetic behavior, and each novel cCPE-based compound should therefore be fully characterized. Some of the disadvantages of our approach include protein production, which was variable between clones and between batches and therefore would benefit from optimization. To improve the pharmacokinetic profile of the new cCPE-based probes, the composition and position of the polyhistidine tag could be changed to further reduce nonspecific uptake (36,37). The selection of a different chelator may also increase tumor-to-background contrast and might be indicated if a shorter-lived radioisotope is chosen in view of the fast pharmacokinetics of the cCPE peptides (38).

The work presented here provides a proof of principle for optimization of cCPE-based imaging agents, based on point mutations of the wild-type sequence. Possible applications include their use as diagnostic tools for PET or SPECT, intraoperative fluorescence imaging agents, or tumor-targeting vectors.

## CONCLUSION

[^111^In]In-cCPE mutants are a useful tool for noninvasive imaging of claudin-4, which is a widely dysregulated and highly prognostic biomarker in pancreatic cancer. These imaging agent could therefore be used to aid in the early detection and characterization of this malignancy, among other applications.

## DISCLOSURE

This research was supported financially by the CRUK/MRC Oxford Institute for Radiation Oncology (Michael Mosley, Veerle Kersemans, and Bart Cornelissen), MRC (Sofia Koustoulidou), Pancreatic Cancer U.K. (Samantha Hopkins), and Pancreatic Cancer Research Fund (Julia Baguña Torres). Apirat Chaikuad and Stephan Knapp receive support from the Structural Genomics Consortium, a registered charity (charity 1097737) that receives funds from AbbVie, Bayer Pharma AG, Boehringer Ingelheim, the Canada Foundation for Innovation, the Eshelman Institute for Innovation, Genome Canada, the Innovative Medicines Initiative (EU/EFPIA), Janssen, Merck KGaA (Germany), MSD, Novartis Pharma AG, the Ontario Ministry of Economic Development and Innovation, Pfizer, the São Paulo Research Foundation-FAPESP, Takeda, and Wellcome. No other potential conflict of interest relevant to this article was reported.

**KEY POINTS**

**QUESTION:** Can optimization of the radiolabeled cCPE protein sequence and structure lead to improved characteristics of this radiopharmaceutical for imaging claudin?
**PERTINENT FINDINGS:** Truncated, smaller, and mutant versions of labeled cCPE demonstrated superior binding affinity and improved pharmacokinetics compared with previously reported compounds.
**IMPLICATIONS FOR PATIENT CARE:** Optimization of the cCPE sequence may lead to improved detection of claudin-4–expressing cancers.
